# Three-dimensional cell culture conditions promoted the Mesenchymal-Amoeboid Transition in the Triple-Negative Breast Cancer cell line MDA-MB-231

**DOI:** 10.3389/fcell.2024.1435708

**Published:** 2024-08-02

**Authors:** Daniela Rodríguez-Cruz, Aleix Boquet-Pujadas, Eunice López-Muñoz, Ruth Rincón-Heredia, Rodolfo Paredes-Díaz, Mauricio Flores-Fortis, Jean-Christophe Olivo-Marin, Nancy Guillén, Arturo Aguilar-Rojas

**Affiliations:** ^1^ Medical Research Unit in Reproductive Medicine, High Specialty Medical Unit in Gynecology and Obstetrics No. 4 “Luis Castelazo Ayala”, Mexican Social Security Institute, Mexico City, Mexico; ^2^ École Polytechnique Fédérale de Lausanne, Biomedical Imaging Group, Lausanne, Switzerland; ^3^ Bioimage Analysis Unit, Pasteur Institute, Paris, France; ^4^ National Center for Scientific Research, CNRS UMR3691, Paris, France; ^5^ Microscopy Core Unit, Institute of Cellular Physiology, National Autonomous University of Mexico, Mexico City, Mexico; ^6^ Cuajimalpa Unit, Engineering and Natural Science Doctoral Program, Metropolitan Autonomous University, Mexico City, Mexico; ^7^ Cuajimalpa Unit, Department of Natural Science, Metropolitan Autonomous University, Mexico City, Mexico; ^8^ National Center for Scientific Research, CNRS ERL9195, Paris, France

**Keywords:** breast cancer, three-dimensional (3D) growth conditions, epithelial-mesenchymal transition (EMT), mesenchymal-amoeboid transition (MAT), metastasis

## Abstract

**Introduction:**

Breast cancer (BC) is the leading cause of death among women, primarily due to its potential for metastasis. As BC progresses, the extracellular matrix (ECM) produces more type-I collagen, resulting in increased stiffness. This alteration influences cellular behaviors such as migration, invasion, and metastasis. Specifically, cancer cells undergo changes in gene expression that initially promote an epithelial-to-mesenchymal transition (EMT) and subsequently, a transition from a mesenchymal to an amoeboid (MAT) migration mode. In this way, cancer cells can migrate more easily through the stiffer microenvironment. Despite their importance, understanding MATs remains challenging due to the difficulty of replicating *in vitro* the conditions for cell migration that are observed *in vivo*.

**Methods:**

To address this challenge, we developed a three-dimensional (3D) growth system that replicates the different matrix properties observed during the progression of a breast tumor. We used this model to study the migration and invasion of the Triple-Negative BC (TNBC) cell line MDA-MB-231, which is particularly subject to metastasis.

**Results:**

Our results indicate that denser collagen matrices present a reduction in porosity, collagen fiber size, and collagen fiber orientation, which are associated with the transition of cells to a rounder morphology with bleb-like protrusions. We quantified how this transition is associated with a more persistent migration, an enhanced invasion capacity, and a reduced secretion of matrix metalloproteinases.

**Discussion:**

Our findings suggest that the proposed 3D growth conditions (especially those with high collagen concentrations) mimic key features of MATs, providing a new platform to study the physiology of migratory transitions and their role in BC progression.

## 1 Introduction

Breast cancer (BC) is the most common global malignant neoplasm in women. In 2020, there were 2.3 million new cases, accounting for 24% of all new cancer cases (Lei et al., 2021; Sung et al., 2021). Mortality rates have also been increasing, with an estimated 680,000 deaths in 2020, representing 15% of cancer-related deaths in the worldwide population (Lei et al., 2021; Sung et al., 2021). Over 90% of BC deaths are caused by metastasis (Gupta and Massagué, 2006). This malignant characteristic is defined as the cell migration through a physical barrier to leave the primary tumor and spread throughout the body to colonize distant organs (Gupta and Massagué, 2006; Cooke et al., 2012; Zheng et al., 2014). The main tissues invaded by metastases in BC are bone, lung, liver, and lymph nodes (Park et al., 2020). These metastatic lesions deteriorate patient health, forming multiple tumor foci which in most cases, are difficult to remove and also are resistant to therapeutic treatments. Thus, understanding and combating metastatic processes have vital importance to counteract the adverse effects of the disease.

Progression toward metastasis is known to be a selective process that involves a multistage model: 1) cell detachment from the primary tumor, 2) cell invasion and migration through the stroma, 3) intravasation into the lymphatic and/or blood vessels, 4) migration through them, 5) extravasation to new sites, and finally, 6) colonization of the new organ (López-Muñoz and Méndez-Montes, 2013; Bhat et al., 2024). The acquisition of these cellular behaviors is known as epithelial-mesenchymal transition (EMT) (Park et al., 2022). The acquisition of EMT requires the conversion from epithelial to mesenchymal cell phenotype by the reconditioning of gene expression (Li et al., 2024). Although EMT is considered the first step of metastasis, cell plasticity may play an important role in the various stages of the metastatic process by facilitating the adaptation of tumor cells to the microenvironment. Therefore, malignant cells must respond to different environments, displaying two migration modes termed mesenchymal and amoeboid (Hecht et al., 2015).

Mesenchymal cell migration has been principally studied in 2D substrates (Hecht et al., 2015; Innocenti, 2018). This migratory mode is prevalent in elongated cells with established polarity and presents a relatively slow migration speed (Bear and Haugh, 2014). During this locomotion, cytoskeletal contractility, adhesion to the extracellular matrix (ECM) via integrins, and enzymatic breakdown of the surrounding matrix by matrix metalloproteinase (MMP) are defining characteristics (O’Neill, 2009). In this case, Focal Adhesion Kinase (FAK) and Src kinases regulate cytoskeleton reorganization and contractility, promoting the formation of focal adhesions to the ECM (Raji et al., 2023). As well, actinin and spectrin play crucial roles in cell migration by maintaining structural integrity and facilitating dynamic changes in cell shape and adhesion. Actinin cross-links actin filaments to stabilize cell structure, while spectrin provides structural support and regulates membrane deformability (Gardel et al., 2010; Thomas et al., 2019). Likewise, syndecans, particularly syndecan-1 (CD138), play crucial roles in cell migration by modulating interaction with several polypeptides, such as extracellular matrix molecules, growth factors and adhesion and migration proteins (Couchman, 2021). CD44, a multifunctional cell surface glycoprotein, similarly influences cell migration through its involvement in adhesion and matrix interactions (Primeaux et al., 2022). Additionally, the induction of actomyosin contractility by RhoA and the turnover of focal adhesions to the ECM are necessary processes (Schaks et al., 2019). During forward movement, Rac-1 induced elongation of cells, formation of migratory actin protrusions, and integrin-mediated adhesion to the ECM generate pulling forces and tension towards the ECM at the leading edge (Ma et al., 2023). Simultaneously, RhoA mediated retraction at the rear diminishes anchorage of the cell to the ECM (Schaks et al., 2019). Likewise, RhoA regulates the actomyosin network through the action of myotonic dystrophy kinase-related CDC42-binding kinase (MRCK), which phosphorylates myosin regulatory light chain (MRLC) to promote actomyosin contractility and cytoskeletal reorganization essential for cell migration (Wilkinson et al., 2005). Finally, remodeling of ECM is dependent on MMPs activity. Those molecules play a crucial role in restructuring the ECM through enzymatic breakdown of its components (Raeeszadeh-Sarmazdeh et al., 2020). Likewise, MMPs functions are carefully controlled through proteolytic activation and inhibition mechanisms involving their endogenous inhibitors known as tissue inhibitors of metalloproteinases (TIMPs) (Coates-Park et al., 2024).

Amoeboid mode of cell migration occurs in rounder cells with weakly defined polarity, low adhesion to the substrate, and high activity levels of the actomyosin cytoskeleton regulated by the small GTPase RhoA and ROCK kinase (Krakhmal et al., 2015; Wu et al., 2021). This motility system has been observed during the migration/invasion of individual cells (Nazari et al., 2023), which presents transient membrane blebs and in some cases a pseudopod at the migration front (Bang et al., 2015; Schick and Raz, 2022). Amoeboid motion results from rapid adaptations to changing extracellular physical environment conditions; it is not based on profound changes in the activity of transcriptional factors (Paňková et al., 2010; Callan-Jones, 2022). It is protease independent where cells are able to rapidly change shape and crawl by means of the blebs through pre-existing spaces in the extracellular matrix (Wu et al., 2021; Chikina et al., 2024).

The study of amoeboid motility during mesenchymal-amoeboid transition (MAT) has been difficult due to a lack of suitable *in vitro* or *in vivo* models. Nevertheless, by high-resolution images techniques it has been observed in *in vivo* models, tumoral cells with amoeboid morphology that move at constant speeds (Wyckoff et al., 2000; Condeelis and Segall, 2003). Several regulating mechanisms seem to promote MAT including the balance between the activity levels of the small GTPases RhoA, Rac, and Cdc42 (Alexandrova et al., 2020), the loss of the ability to degrade the ECM (Wolf et al., 2003) and the density or architecture of extracellular environment (Ehrbar et al., 2011; Glentis et al., 2017; Velez et al., 2017). This last point is highly relevant during BC metastasis development considering several observations. First, notable alterations occur in the composition of ECM, with an elevated production of type-I collagen being recognized as a particularly significant change (Insua-Rodríguez and Oskarsson, 2016). For example, while the collagen concentration in normal tissue typically is around 2.5 mg/mL, in advanced stages of BC, tumors exhibit levels as high as 6 mg/mL (Kumar and Weaver, 2009; Fraley et al., 2015). As well, there is a direct correlation between the greater number of regions with high collagen concentration and the aggressiveness increase of various solid tumors, including breast tumors (Provenzano et al., 2008). The above information suggests that extreme dense environments can promote the selection of more aggressive tumor cells. Finally, the topography of collagen fibers arrangement in these high-density areas has been correlated with tumor progression and metastasis (Conklin et al., 2011; Morkunas et al., 2021; Sprague et al., 2021). These data suggest that the environment with high concentrations of ECM fibers also promotes migration and invasion, indicating that the mechanisms by which the increase in the stiffness of the ECM promotes MAT and metastasis deserve attention in cancer research.

To investigate the impact of denser environments on cell migration and invasion, Triple-Negative BC (TNBC) cells, MDA-MB-231, were seeded into increasing collagen concentrations within a three-dimensional (3D) growth environment. Using laser two-photon microscopy, image analysis, and MMP secretion measurements, we determine that higher stiffness levels had a significant effect on the cell migration characteristics correlated with induction of a rounder cell morphology with bleb-like extensions. These cells display more persistent migration, improve their invasion capacity on collagen, and an unexpected reduction in MMPs secretion, alongside an increase in TIMP-1 and TIMP-2. Based on this data, we propose that the 3D growth conditions presented here can mimic important aspects observed *in vivo* in advanced stages of BC, such as a high amount of collagen, and the presence of a small population of cells with the capacity to undergo MAT, providing a new platform to study the physiology of migratory transitions and their role in BC progression.

## 2 Materials and methods

### 2.1 Cell culture in two-dimension (2D) conditions

The human mammary cancer cell line MDA-MB-231 (HTB-26, ATCC, USA) was employed as model. This aggressive cell line exhibits a TNBC phenotype and has undergone EMT (Fillmore and Kuperwasser, 2008). MDA-MB-231 cells were maintained in 1X Leibovitz’s L-15 medium (Gibco, USA), supplemented with 10% fetal bovine serum (FBS) (Invitrogen, USA), and an antibiotic mixture (Gibco, USA). The growth temperature was at 37°C in an atmosphere of 5% CO_2_ and 95% O_2_.

### 2.2 Three-dimensional (3D) growth culture conditions

Type-I collagen purified from rat tail (Corning, EUA) was used in a range from 1 mg/mL, 3 mg/mL and 6 mg/mL. 3D cells growth conditions were elaborated through our method. Briefly, the model base is a collagen solution in which the alkaline pH is inactivated, promoting its polymerization to gel. To ensure the quality and reproducibility of all the gels used in each assay, they were all prepared using 1 mL of stock solution. Then, from this stock solution, a specific volume was added to the appropriate well or plate, depending on the assay to be performed. Additionally, conditions such as pH value, ionic strength, time, and temperature for polymerization were kept uniform across all assays. The solution of collagen mix was constituted by 1X Leibovitz’s L-15 medium (Gibco), 10% FBS (Invitrogen) and the volume of rat collagen to obtain the intended concentration (1 mg/mL, 3 mg/mL, or 6 mg/mL) (Corning USA). The pH in the solution was neutralized with the addition of 0.1 N NaOH (Sigma-Aldrich, USA). Afterwards, MDA-MB-231 cells were added to the mixture. The solution was mixed carefully and kept at 37°C for 40 min to promote polymerization. Finally, as previously shown (Velez et al., 2017), all microscopy images were captured in the center of the collagen matrices and at an appropriate distance from the bottom of the slides to avoid any influence on the cells. Similarly, all the images considered only cells that were completely embedded in the collagen matrices.

### 2.3 Evaluation of cellular proliferation in 3D growth conditions

To assess the impact of increased collagen concentration on the proliferation of the MDA-MB-231 cell line, the CellTiter-Glo 3D Cell Viability Assay kit was used following the manufacturer instructions (Promega, USA). Briefly, 100 µL of collagen gels at concentrations of 1 mg/mL, 3 mg/mL, and 6 mg/mL were prepared as described above and added into 96 well-plate (Corning, USA). In the mixture of each condition, 20,000 cells were added per well. The 3D systems were cultured for 24 h (Time 1, T1), 48 h (Time 2, T2), and 72 h (Time 3, T3). As a baseline reference, evaluation was conducted two hours after seeding, denoted as time 0 (T0). Following each time point, CellTiter-Glo 3D reagent which measures ATP as an indicator of viability was added to induce cell lysis. Then, luminescence was recorded using a Synergy-HTX microplate reader (Biotek, USA), with an integration time of 0.75 s per well. Cell proliferation was quantified as Relative Luminescence Units (RLU) ± Standard Deviation (SD). All assays were performed in triplicate, and statistical differences were assessed using Prism 8 software (GraphPad, USA).

### 2.4 Evaluation of cellular viability in 3D growth conditions

Cell death at different concentrations of collagen was measured by double staining with CellTracker Green (Invitrogen) and propidium iodide in red (Sigma Aldrich). In brief, cultured MDA-MB-231 cells were labeled with the fluorescent compound CellTracker Green, in accordance with the protocol provided by the manufacturer (Invitrogen). The labeled cells were then recovered and enumerated. Then, in 35 mm glass-bottom culture dishes (Ibidi, Germany), 500 μL of collagen prepared as above mentioned and at concentrations of 1 mg/mL, 3 mg/mL, or 6 mg/mL, containing 250,000 cells per condition, were placed, and maintained for 24 h under optimal growth conditions. At the end of each time point, dead cells were stained with propidium iodide and then rinsed twice with 1X PBS. Cell fixation was performed using 3.7% paraformaldehyde (PFA) (Sigma Aldrich). All samples were mounted with ProLong glass antifade (ThermoFisher Scientific, USA).

Cells from each time point were subjected to visualization by fluorescence microscopy using a standard TRITC filter and FITC filter in LSM800 laser-scanning microscope (Zeiss, Germany). Z slices were acquired every 0.5 μm over a total distance of 100 μm in Z. The acquisition of images was realized under the following conditions: image size (pixels) 1024 × 1024, a bit depth of 16 bits, a Plan-Apochromat 10×/0.45. The lasers used were 562 nm with 1 AU and 0.3% of laser potency and 488 nm with a 0.98 AU and 3% of potency. All the images were acquired on frame and unidirectional mode, occupy 300–450 μm of thickness and ×10 water-immersion objective with a numerical aperture of 0.45 (Zeiss). Control of the microscope and laser was facilitated by the Zen 2011 SP7 software package (Zeiss). The images were then visualized and both channels were superimposed using the Icy software (*n* = 4 fields for sample) (http://icy.bioimageanalysis.org) (De Chaumont et al., 2012). Cells highlighted in yellow by the superposition of both channels, was indicative of cell death. In brief, in 4 fields of each sample, 100 cells were counted randomly in the green channel. It was considered as 100%. Then, in the same fields, were counted the number of cells stained in the red channel. It was considered as the percentage of cell death with respect to the total cells and reported as a percentage of cell viability. Superposition of both channels and quantification of yellow bright plots was carried out in the mentioned software. All assays were performed in triplicate, and statistical differences were assessed using Prism 8 software (GraphPad, USA).

Cells undergoing apoptosis were examined using the CellEvent Caspase‐3/7 Detection Reagent (Green) (Invitrogen), following the manufacturer instructions. In brief, cell batches were prepared at the three collagen concentrations following the method mentioned above. Subsequently, all of them were incubated for 24 h under regular growth conditions. Then, a 10× staining solution (Invitrogen), was added at a 1:100 dilution and kept in a cell culture hood for 30 min. Cell nuclei were stained with 300 nM DAPI stain solution (Invitrogen) and fixed with 3.7% PFA (Sigma Aldrich) for 1 h. All samples were mounted with ProLong glass antifade (ThermoFisher Scientific) and visualized in an LSM800 laser-scanning microscope (Zeiss) using the FITC and DAPI filters. Z slices were acquired every 0.5 μm over a total distance of 100 μm in Z. The acquisition of images was realized under the following conditions: image size (pixels) 1024 × 1024, a bit depth of 16 bits, a Plan-Apochromat 10×/0.45. The lasers used were 488 nm with a 0.98 AU and 3% of potency, and 405 nm with 1 AU and 0.3% of laser potency. All the images were acquired on frame and unidirectional mode, occupy 300–450 μm of thickness and ×10 water-immersion objective with a numerical aperture of 0.45 (Zeiss). Control of the microscope and laser was facilitated by the Zen blue 2.6 edition software package (Zeiss). Superimposition of both channels was carried out in the mentioned software (De Chaumont et al., 2012). Additionally, quantification of cells undergoing apoptosis after 24 h was determined by the bright blue signal in 4 fields (n:100 cells), as was mentioned above, using the Icy software. All assays were performed in triplicate, and statistical differences were assessed using Prism 8 software (GraphPad).

### 2.5 Evaluation of cytoskeletal and morphological changes in 3D growth conditions

To determine morphology changes, cells were visualized using confocal microscopy, following procedures described elsewhere (Geraldo et al., 2013). In brief, 500 μL of collagen at concentrations of 1 mg/mL, 3 mg/mL, or 6 mg/mL, each containing 250,000 MDA-MB-231 cells, were seeded onto 35 mm glass-bottom culture dishes (Ibidi). Each cell batch was then incubated under regular growth conditions for 24 h and fixed and extracted, with a solution containing 4% PFA, 0.3% Triton X-100 (Sigma Aldrich), and 5% sucrose (Sigma Aldrich) for 5 min.

F-actin was stained by incubating the cells with 0.165 mM rhodamine-conjugated phalloidin (Invitrogen, Molecular Probes, USA) overnight in the dark at room temperature. Cells nuclei were stained with 300 nM DAPI solution as described above and mounted with ProLong solution (Invitrogen). Samples were then visualized in an LSM800 laser-scanning microscope (Zeiss) using the TRITC and DAPI filters. Z slices were acquired every 0.5 μm over a total distance of 50 μm Z. The acquisition of images was realized under the following conditions: image size (pixels) 1024 × 1024, a bit depth of 16 bits, a Plan-Apochromat 10×/0.45. The lasers used were 532 nm with 1 AU and 0.3% of laser potency and 405 nm with a 1 AU and 3% of laser potency. All the images were acquired on frame and unidirectional mode, occupy 300–450 μm of thickness and ×40 water-immersion objective with a numerical aperture of 1.3 (Zeiss). Control of the microscope and laser was facilitated by the Zen blue 2.6 edition software package (Zeiss). We used the “3D VTK” plugin in Icy software (De Chaumont et al., 2012), to reconstruct the cells in 3D and employed the “3D ruler helper” to measure the longitudinal distance (between the furthest points) and the transversal distance of the cells (between the furthest points) (1 mg/mL *n* = 11, 3 mg/mL *n* = 10 and 6 mg/mL *n* = 5). We also computed the volume of each cell. For this, we independently segmented the two channels (F-actin and nuclei) of each cell using an HK-means hierarchical algorithm (3 classes, with a minimum region size of 10,000 voxels). We used both channels because the F-actin channel marked the outside very well, while the inside of the cell was better covered by the nucleus signal. We then filled the holes in the resulting masks (one per channel) and computed the union between the two. The final union “mask” yielded a good segmentation of the cell, and we translated the number of voxels of the mask into μm^3^ using the metadata. This segmentation process was also performed in Icy software (De Chaumont et al., 2012).

### 2.6 Evaluation of cellular morphology in 3D growth conditions

To further evaluate morphology of the cells, Transmission Electron Microscopy (TEM) was performed. To do this, 100 μL of collagen at concentrations of 1 mg/mL, 3 mg/mL, or 6 mg/mL, containing 150,000 cells per concentration, were grown on 96 well-plates (Corning). All samples were fixed with 1.0% glutaraldehyde (Sigma Aldrich) in PBS buffer at pH 7.2 for 2 h at 4°C then, after a PBS wash, they were subjected to postfixation with 1% osmium tetroxide (Sigma Aldrich) for 2 h at 4°C. Subsequent steps involved rinsing and dehydration in a sequence of ethanol concentrations, culminating in acetonitrile (Sigma Aldrich). Pre-inclusion was accomplished by incubating the samples in a mixture of acetonitrile and Epon resin at a 1:1 ratio for 48 h. Finally, the samples were embedded in Epon resin and left to cure for 48 h at 60°C. The contrast of the samples was carried out with uranium acetate and lead nitrate. Thin sections of longitudinal and transverse views of MDA-MB-231 cells, with approximately 80 nm in thickness, were prepared using a Reichert Jung microtome (Austria) (Spector et al., 1998) and observed using a JEOL JEM-1200 EX II scanning electron microscope (*n* = 3 for each condition) (Peabody, USA).

### 2.7 Evaluation of cellular migration in 3D growth conditions

To evaluate the migration ability of MDA-MB-231 cells in 3D growth conditions, we used a lens-free microscope. This setup allows the continuous monitoring of cell migration inside an incubator at a controlled temperature and humidity, without any modifications in cell integrity or cell environment (Kim et al., 2012). MDA-MB-231 cells were grown in 2D conditions in 6 well-plates (Corning). Sixteen hours prior to the assay, the cells were serum-starved in Leibovitz’s serum-free medium to promote better responses during the cell migration assay. Starved cells were recovered, counted and seeded in 3D conditions. All the assays were carried out in 27 mm glass-bottom culture dishes (Thermo Fisher). In order to prevent focal plane loss, all recordings were taken in dishes coated with a thin layer of collagen. Over this layer, MDA-MB-231 cells were seeded and covered with a thicker layer of collagen. To maintain the stiffness conditions in the system, both the upper and lower layers of collagen had the same concentration. To promote cell migration, 10% FCS was used as chemoattractant agent, which was contained within each collagen gel.

Before the assay, MDA-MB-231 cells were maintained in fasting conditions for at least 16 h before starting the assay to promote cell migration. On the day of the assay, culture dishes were covered with 200 μL of collagen at 1 mg/mL, 3 mg/mL, or 6 mg/mL prepared as mentioned in Section 2.2. After collagen polymerization into a gel, cells were counted, and 20,000 cells were seeded. Then, the system was covered with free-serum Leibovitz’s L-15 media for 45 min in a cell culture hood to promote cell adhesion. After this time, the media was removed and 350 μL of collagen at 1 mg/mL, 3 mg/mL, or 6 mg/mL was added to each plate as a thicker layer to evaluate the cell migration in 3D conditions. To promote collagen gelation, all the samples were incubated at 37°C for 40 min. Finally, the system was covered with free-serum Leibovitz’s L-15 and maintained in growth conditions for 24 h. Cell migration recording was performed during this period with a lens-free video microscopy equipment that uses the in-line holographic imaging method. It comprises a 12-bit APTINA MT9P031 CMOS RGB imaging sensor with a pixel pitch of 2.2 μm, measuring 5.7 × 4.3 mm, and a light-emitting diode (LED) with a dominant wavelength of 525 nm, accompanied by a pinhole of 150 μm, as previously reported (Kesavan et al., 2014). The lens-free video-microscope was positioned in the culture hood, and the petri dish containing the cells was placed inside.

Long-term videos of the cells on the focal plane were acquired with this setup to allow for live imaging while maintaining optimal growth conditions throughout the entire duration of the experiment. Videos were recorded for 24 h with a frame interval of 10 min. This interval was chosen in accordance with previous reports carried out with lens-free microscopy and breast cancer cells (Mathieu et al., 2016). The magnification was low due to the lensless setup chosen to ensure optimal growth conditions. This made the movement of protrusions difficult to monitor, so we focused on tracking the migration of entire cells. To examine the migration of the cells quantitatively (Boquet-Pujadas et al., 2021), we tracked their trajectories through the ECM using TrackMate software (*n* = 135 at 1 mg/mL, *n* = 81 at 3 mg/mL and *n* = 71 at 6 mg/mL) (Tinevez et al., 2017). Specifically, the videos were first preprocessed with a max filter of size 2. The cells were detected with a Laplacian of Gaussian filter at each frame (parameters in TrackMate: 0.3 threshold, 50 μm blob diameter, no median filter, no subpixel localization). They were then tracked by solving linear-assignment problems to link the cells between one frame and the next and to close any gaps (parameters in TrackMate: 73.4 max distance, 57.1 max gap distance, 10 max frame gap, no feature penalties). The tracking was semi-automatic; we corrected it manually by iterating through the interactive interface in TrackMate. The resulting tracks were analyzed in terms of four different measures using a custom-made script in R. The measures were i) the mean (over all frames) of the instantaneous speed of the cells between consecutive frames; ii) the percentage of static cells, i.e., the proportion of cells that moved a total distance smaller than 1.5 times the diameter of the cell (i.e., 75 μm) over the acquisition period. Note that the percentage of static cells indicates the proportion of cells that do not migrate, possibly because they are stuck in the matrix.; iii) the α-value, which was computed as the slope of the log-log plot of the mean-squared displacement (MSD) of the trajectory of each cell over increasing time intervals; and iv) the persistence, which was computed as the ratio of net displacement to total displacement of the cell.

### 2.8 Evaluation of cellular invasion of collagen in 3D growth conditions

MDA-MB-231 cells were maintained in 2D culture under regular conditions in 6 well-plates (Corning). Sixteen hours prior to the assay, the cells were serum-starved in Leibovitz’s serum-free medium to promote cell invasion and then stained with CellTracker Red (Invitrogen), following the manufacturer instructions. Stained cells were recovered and counted. One milliliter of collagen at 1 mg/mL, 3 mg/mL, and 6 mg/mL concentrations prepared as was described in Section 2.2 was loaded in 35 mm glass-bottomed dishes. This volume allows obtaining collagen cubes with a focal distance of ∼1,000 μm in depth. Upon these supports, 100 μL of serum-free medium containing 120,000 labeled cells were placed. To promote cell adhesion, the hanging supports with the cells were kept in the culture incubator for 40 min. Then, the system was overlaid with two milliliters of serum-free medium. To promote cell invasion, a chemoattractant agent consisting of 10% FCS was employed inside each collagen cube. Invasion of the collagen supports was allowed to proceed for 24 h under optimal culture conditions. At the conclusion of this period, the device was washed with 1× PBS and fixed with 4.0% PFA (Sigma Aldrich) in PBS for 1 h.

Collagen structure and cell invasion was evaluated using multiphoton microscopy employing an LSM710 NLO laser-scanning microscope (Zeiss) coupled with a TI:sapphire femtosecond laser (Chameleon ultra II; Coherent) as previously reported (Aguilar-Rojas et al., 2020). To capture the entire depth of collagen cubes (∼1,000 μm), a Plan Apochromat ×20 water-immersion objective with a numerical aperture of 1 (Zeiss) was utilized. Water-based ultrasonic couplant was employed as the mounting media due to its refractive-index-matching properties with water. The emission laser wavelength utilized for excitation was 820 nm. Signals from the collagen matrix were captured by second harmonic generation (SHG) technique. This approach is highly sensitive and can reveal critical biological information about ECM changes that occur during breast cancer progression and metastasis (Ambekar et al., 2012). For this, we employed a non-descanned detector (NDD) with a bandwidth filter spanning 395–425 nm in a backscattering geometry. To evaluate cell migration, labeled cells were detected using CellTracker Red (Invitrogen) with two-photon laser scanning microscopy. The detection bandwidth ranged from 570 to 610 nm, pseudo colored in red. Image acquisition of X and Y planes was performed with a frame size of 512 × 512 pixels and a pixel size of 0.42 μm × 0.42 μm. Images were captured at various depths with a Z step of 10 μm. Control of the microscope and laser was facilitated by the Zen black software package from Zeiss.

The evaluation of collagen fibers was manually carried out with the “ruler helper” tool of Icy software as previously described (Karamikamkar et al., 2018). Briefly, three layers were selected, one from the top, one from the middle, and one from the bottom of each collagen condition. Each image was converted to 8-bit format, followed by adjusting the threshold until the desired area appeared against a black and white background. The threshold value was kept consistent across images from different concentrations. This approach enabled a comparative analysis of all specimens and facilitated fiber identification. Once the images were prepared for analysis, twenty fibers were randomly selected in each layer, and the “ruler helper” tool was used to measure the length of the fibers. Additionally, free-space length among the fibers was determined following the method previously described (Karamikamkar et al., 2018). Free-space domains without collagen fibers were found and defined as the Lf value. To determine the Lf value, one layer of SGH images positioned at the top, one in the middle, and one at the bottom for each collagen concentration were converted to black and white bitmaps to distinguish collagen fibers from the background. Then, the largest square areas that were free of black pixels were defined and measured. This method was applied to SGH images of each collagen concentration and repeated 20 times for each sample. Likewise, this evaluation finds the largest square for which the most probable number of crossing black pixels in a randomly placed square is zero. Variations in Lf parameters were interpreted in the context of collagen concentration, considering that the Lf value is reduced as the concentration is increased and fiber size is also reduced (Karamikamkar et al., 2018).

To evaluate the structural organization and properties of collagen fibers at different concentrations, Fast Fourier Transform (FFT) was carried out as described previously (Tang et al., 2014; Brisson et al., 2015; Pijanka et al., 2019). In brief, SHG microscopy images were imported into ImageJ software for preprocessing. One stack with twenty layers of specific Regions of Interest (ROIs) (layers from 50 to 70 in each case), containing well-defined collagen structures, was selected for further analysis. To enhance the clarity and contrast of collagen fibers, brightness and contrast adjustments were applied, and noise reduction filters were utilized as necessary. The percentage of SHG-positive pixels within each ROI was calculated using ImageJ software. This metric served as a quantitative measure of collagen fiber intensity. Then, ROIs were subjected to FFT using ImageJ’s FFT function. The resulting FFT power plots were generated to visualize the distribution of spatial frequencies within the collagen fibers. Intensity of pixels in the black area of each image was derived from the FFT power plots and, pore size and fiber size, were extracted and analyzed. The calculated pore size and fiber size for different collagen concentrations were compiled and analyzed to assess the structural organization of collagen. Variations in these parameters were interpreted in the context of collagen concentration, providing a comprehensive understanding of how collagen structure changes with concentration.

Finally, to measure the distance traveled by each cell, we used the Icy software (De Chaumont et al., 2012) to compute 3D projections of each collagen cube containing labeled cells in red. For this, SHG images were converted to 8-bit format, and subjected to threshold adjustments to highlight the positions of each cell. The threshold value was maintained consistently across images from various concentrations, enabling a comparative analysis of all specimens. The distance traveled by each cell under all conditions was measured from the top of the gel to the cell position using the “3D ruler helper” tool in Icy software. The lengths of the distances traveled were expressed in micrometers using the metadata. Then, the total distance was divided into quartiles with a length of 250 μm each one. The number of cells in each quartile was determined. To minimize errors arising from potential differences in total distances of the collagen gels, the number of cells per quartile was quantified considering 750 μm as the maximum distance in each condition.

### 2.9 Evaluation of collagen degradation in 3D growth conditions

To determine changes in the ability of MDA-MB-231 cells to degrade collagen in response to increased concentrations of collagen, degradation of DQ-FITC-labeled type-I collagen monomers (Invitrogen, Molecular Probes) was carried out with slight changes to the protocol previously reported (Thibeaux et al., 2014). In brief, Collagen solutions at concentrations of 1 mg/mL, 3 mg/mL, or 6 mg/mL were prepared according to the method outlined in Section 2.2 and enhanced with 2% DQ-FITC-labeled type-I collagen monomers (collagen-DQ solution) (Invitrogen, Molecular Probes). Subsequently, 100 μL of each concentration of collagen-DQ solution was dispensed into a 96-well plate (Corning) containing 150,000 MDA-MB-231 cells recovered after growth in regular conditions. Then, each sample was incubated under optimal growth conditions for 24 h. Following incubation, solid-phase collagen was centrifuged for 5 min at 16,000 g (Eppendorf, Germany), and the resultant supernatant containing released fluorescein isothiocyanate–collagen fragments was quantified using the Synergy-HTX microplate reader (Biotek) with excitation at 485 nm and detection at 520 nm.

Background was detected in samples without cells, and total degradation was recorded in the presence of type-VII collagenase from *Clostridium histolyticum* (7 U/mL, for 6 h, at 37°C) (Thibeaux et al., 2014). Three independent experiments were performed. Data are reported as the percentage of degradation of type-I collagen relative to total degradation, with SD indicated. All assays were conducted in triplicate, and statistical differences were assessed using Prism 8 software (GraphPad).

### 2.10 Evaluation of MMP/TIMP secretion in 3D growth conditions

To assess the impact of different collagen concentrations on the secretion of matrix metalloproteinase (MMP) and tissue inhibitor of metalloproteinase (TIMP) in MDA-MB-231 cells, the Human MMP Antibody Array-Membrane (Abcam, USA) was utilized according to the manufacturer instructions. In brief, 100 μL of each collagen solution was dispensed into individual wells of a 96-well plate (Corning), with each well containing 150,000 cells. Upon gel formation, the samples were then incubated under optimal growth conditions for 24 h. After the incubation period, the samples were centrifuged for 5 min at 16,000 g (Eppendorf). The supernatant, containing released proteins, was quantified using the Bradford method. Subsequently, 200 μg/mL of total protein was employed in the Human MMP Antibody Array-Membrane (Abcam) following the manufacturer instructions.

### 2.11 Statistical analysis

All data were analyzed using Prism 8 software (GraphPad) and are presented as mean ± SD. Each data point represents the average of at least three independent experiments. Statistical comparison between two groups was conducted using an unpaired Student’s t-test, with significance defined as *p* < 0.05. Statistical analysis to determine structural differences among all the collagen conditions were carried out by Games-Howell test (*p* < 0.05) and Wilcoxon test (*p* < 0.05).

## 3 Results

### 3.1 Evaluation of 3D growth conditions

**FIGURE 1:**
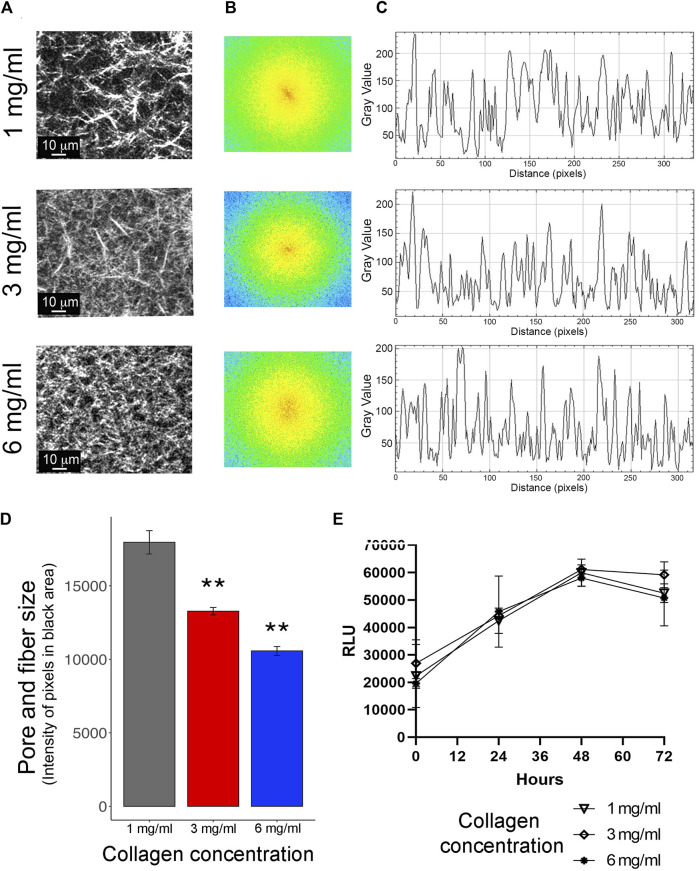
Evaluation of cellular growth in 3D conditions. **(A)** Representative images of collagen matrices at 1 mg/mL, 3 mg/mL, and 6 mg/mL captured by multiphoton microscopy and second harmonic generation (SHG) technique. Scale bar: 10 μm. **(B)** The increase in stiffness with higher concentrations of collagen was evaluated using Fast Fourier Transform (FFT) analysis in ImageJ software. **(C)** Power plots for 1 mg/mL, 3 mg/mL, and 6 mg/mL of collagen were obtained from the FFT test. **(D)** The intensity of pixels in the black area of each power plot was extracted, analyzed, and expressed as pore and fiber size. These analyses were carried out using ImageJ software. **(E)** Evaluation of cellular proliferation of MDA-MB-231 cells embedded in collagen at 1 mg/mL, 3 mg/mL, and 6 mg/mL, measured as Relative Luminescence Units (RLU). Proliferation was assessed at T0 (0 h), T1 (24 h), T2 (48 h), and T3 (72 h). All experiments were conducted in triplicate.

The impact of increasing ECM stiffness on cell migration and invasion was evaluated during the 3D growth of TNBC cells, MDA-MB-231. Type-I collagen as the ECM was employed at diverse concentrations chosen to replicate the proportions found in normal breast tissue (1 and 3 mg/mL) and breast malignant tumors (6 mg/mL) (Fraley et al., 2015; Velez et al., 2017). Visualization of the collagen matrix by two-photon laser scanning microscopy and SHG signal revealed the topology of the ECM. As a consequence of higher collagen concentration, there was a reduction in the length of collagen fibers and the free-space domains among the collagen fibers, referred to as the Lf value (Figure 1A). At 1 mg/mL of collagen, the average fiber extension was 33 μm and the Lf value was 146 μm. Both distances were reduced at 3 mg/mL, showing 16 μm for the fiber length (*p* = 5.31E^−05^) and Lf value of 64 μm (*p* = 1.16E^−05^). Finally, in the case of collagen at 6 mg/mL, the longitudinal extension of the fibers was also reduced to 8 μm (*p* = 1.07E^−06^) and the Lf value was drastically reduced to 11 μm (*p* = 7.05E^−09^) (Figure 1A). Our data show a reduction in collagen fiber length and spaces among the collagen fibers. However, analysis of LF value in collagen structure studies has the disadvantages of dependency on signal quality and misrepresentative results when collagen fibers have complex or overlapping orientations. For these reasons, complementary methods were carried out to analyze collagen structures.

To explore the characteristics of collagen substates, we carried out an FFT analysis of SGH images at collagen concentrations of 1 mg/mL, 3 mg/mL, and 6 mg/mL (Figure 1A). In this analysis, a single-dimension FFT algorithm decomposes the signal into its component frequencies. The shape of the spatial distribution of these frequencies, in this case the orthogonal and planar directions of collagen fibers, is plotted and projected into an elliptical shape. In this context, it is recognized that an ellipse can be related to aligned and oriented collagen fibers, while the loss of the elliptical shape can be associated with randomly oriented collagen fibers (Frisch et al., 2012). In the images at 1 mg/mL of collagen, a local linear aggregation of collagen fibers was observed. This alignment was corroborated by FFT analysis, which shows the first order as an ellipse in the center (Figure 1B, top panel). In contrast, at 3 mg/mL and 6 mg/mL of collagen, no ellipse was observed in the center of the FFT analysis (Figure 1B, middle and bottom panels). This data suggests that higher concentrations promote irregular and stiff collagen fiber alignment. Likewise, the FFT test produced power plots for 1 mg/mL, 3 mg/mL, and 6 mg/mL of collagen (Figure 1C). The intensity of pixels in the black area of each power plot was extracted and analyzed (Figure 1D), showing a clear reduction in pore size and fiber size as collagen concentration increased. All these results show that an increase in collagen concentration promotes disruption in the alignment and length of collagen fibers. Additionally, these data support the idea that there is a positive correlation between polymer concentration and the stiffness of the gel, as previously has been shown (Antoine et al., 2015; Sarrigiannidis et al., 2021).

**FIGURE 2:**
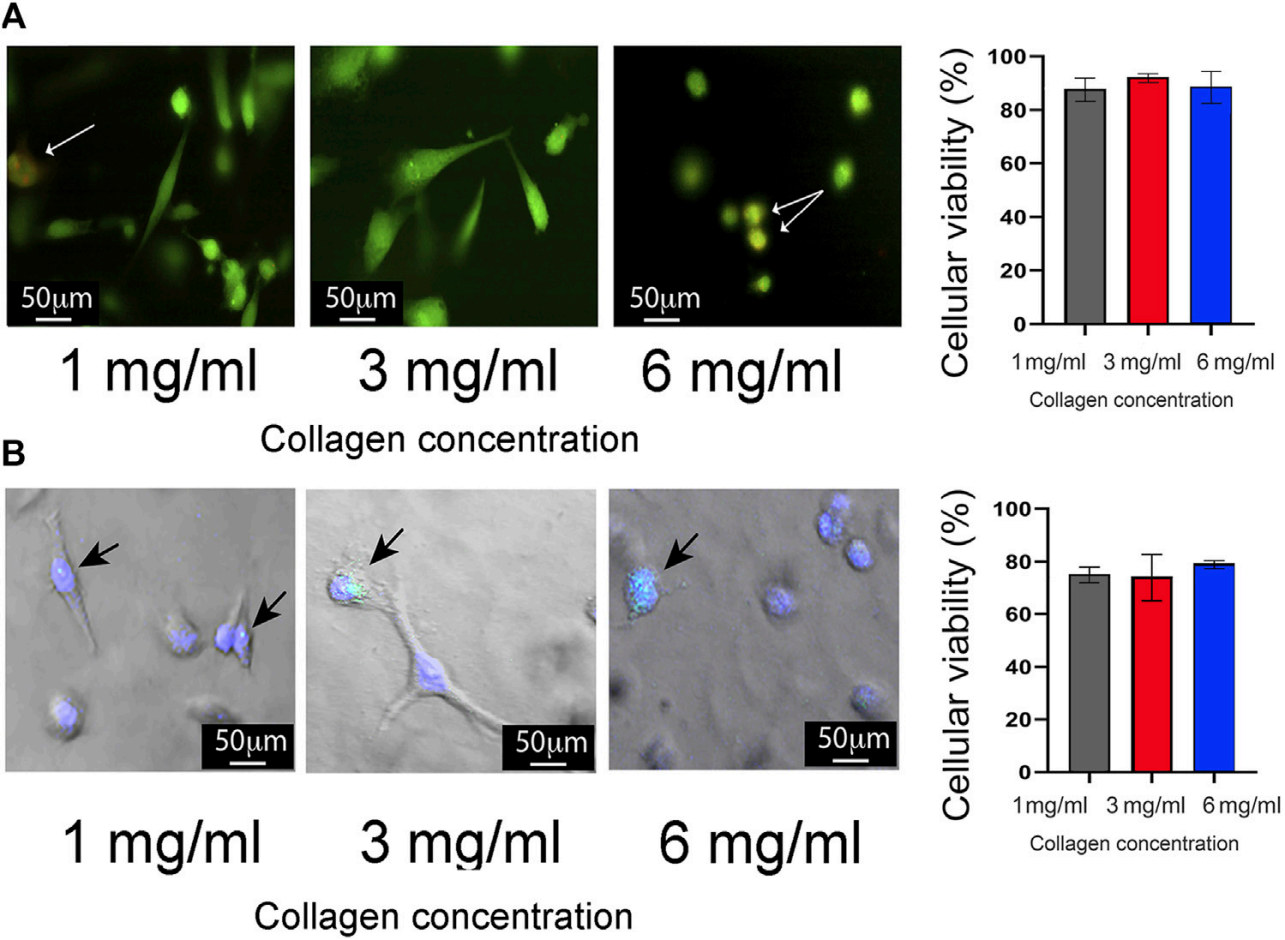
Evaluation of cell viability in 3D growth conditions. **(A)** Left panel: Representative images describing cell viability using double staining with CellTracker Green (green cytoplasm) and propidium iodide (red nucleus). MDA-MB-231 cells were cultured at 1 mg/mL, 3 mg/mL, and 6 mg/mL of collagen. Images were captured using laser scanning confocal microscopy. Cell death is indicated by yellow dots (white arrows), while live cells are shown in green. Scale bar: 50 μm. Right panel: Quantification of cell viability presented as percentage ±standard deviation (SD). **(B)** Left panel: Representative images showing cell death through apoptosis staining with the CellEvent Caspase-3/7 Detection Reagent (green). Apoptotic cells are highlighted by bright blue dots (black arrows), while live cells are visualized by nuclei stained with DAPI stain solution. Scale bar: 50 μm. Left panel: Quantification of apoptotic cell death presented as percentage ± SD. All experiments were conducted in triplicate.

To validate the 3D growth conditions, the proliferation rate of MDA-MB-231 cells was assessed for 72 h. These assays showed consistent cell growth until the first 48 h for 1 mg/mL, 2 mg/mL, and 6 mg/mL of collagen. Subsequently, at 72 h, a reduction in proliferation was observed (Figure 1E). Considering these findings, all subsequent assays were carried out at 24 h post-seeding. Cell cytoplasm and nucleus staining validate MDA-MB-231 cells viability under the three collagen conditions, with 90% survival in all conditions after 24 h of seeding (Figure 2A). Staining for Caspase-3/7 activity evaluated cell apoptosis showing 80% cell viability after 24 h of seeding (Figure 2B). Based on this data, there is a proportion nearly to 20% of MDA-MB-231 cells undergoing necrosis or apoptosis after 24 h of growth in all the 3D conditions. Likewise, the evaluation of cell morphology in the acquired micrographs reveals a substantial morphological change in the cells as the collagen concentration increases. Specifically, at high concentrations of collagen, the cells exhibited a rounder shape, whereas at low concentrations of collagen, the cells show polarized morphology (Figures 2A, B).

### 3.2 Evaluation of cellular morphology in 3D growth conditions

**FIGURE 3:**
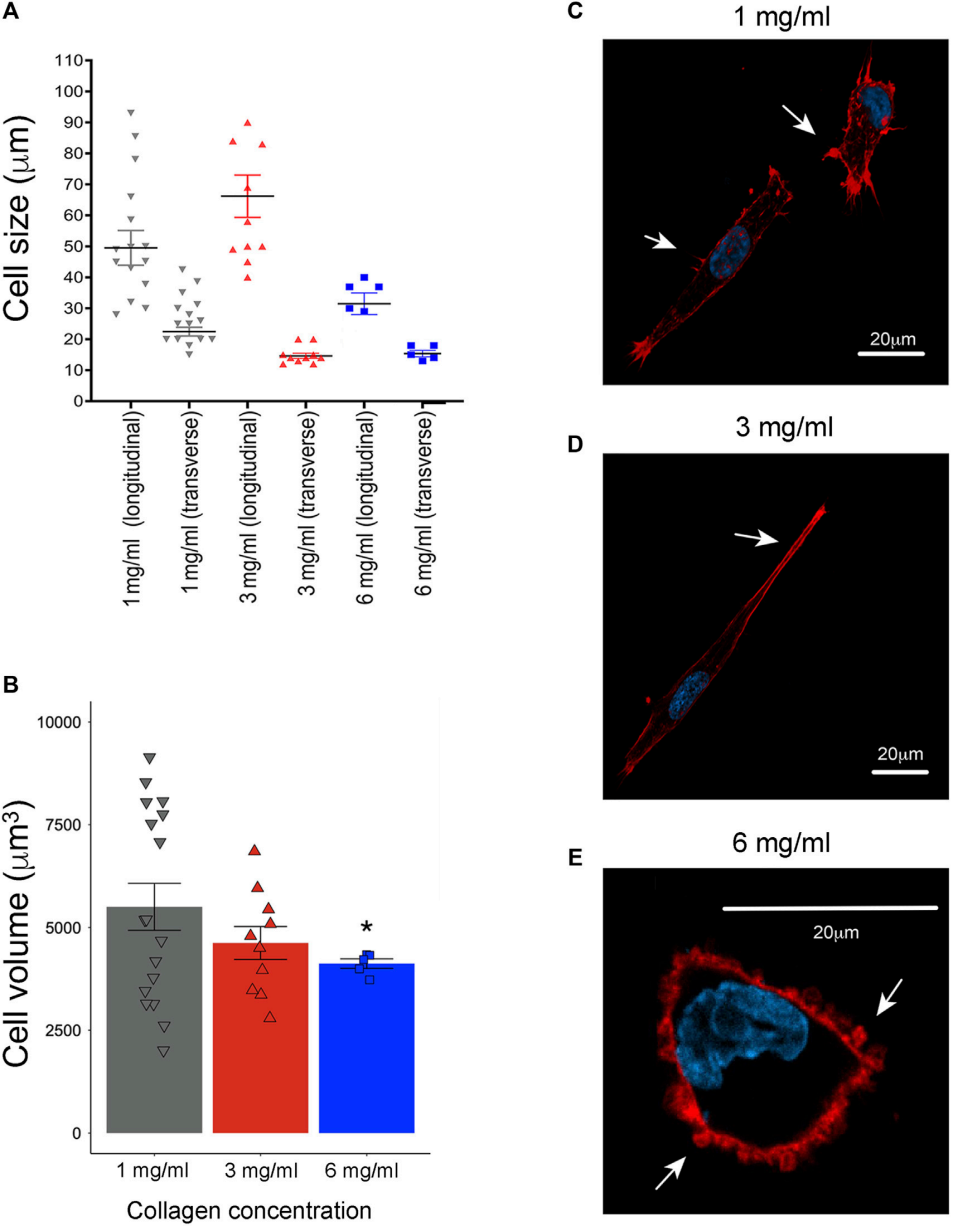
Evaluation of cell morphology in 3D growth conditions by laser scanning confocal microscopy. **(A)** Evaluation of longitudinal axis and transverse axis at 1 mg/mL (averages of 49 μm and 22 μm, respectively), at 3 mg/mL, (average of 66 μm and 14 μm) and 6 mg/mL of collagen (average of 31 μm and 15 μm). All the distances were evaluated with the “ruler helper” plugin using the Icy software (1 mg/mL *n* = 11, 3 mg/mL *n* = 10 and 6 mg/mL *n* = 5) (De Chaumont et al., 2012). **(B)** Evaluation of cellular volume at 1 mg/mL (5,823 μm), 3 mg/mL (4,622 μm) and 6 mg/mL (4,120 μm). F-actin and nuclei channels were independently segmented using an HK-means hierarchical algorithm. This segmentation process was also performed in Icy software (1 mg/mL *n* = 11, 3 mg/mL *n* = 10 and 6 mg/mL *n* = 5) (De Chaumont et al., 2012). **(C)** Representative images of MDA-MB-231 cells cultured at 1 mg/mL of collagen. **(D)** At 3 mg/mL of collagen concentration, and **(E)** at 6 mg/mL of collagen. Notice elongate cell shape with prominent membrane extensions (white arrows) at 1 mg/mL of collagen. Likewise, at 3 mg/mL of collagen was observed an increase in elongate shape with reductions in cell membrane extensions (white arrows). Cells cultured at 6 mg/mL of collagen exhibit a rounded shape and bleb-like as membrane extensions (white arrows). F-actin was stained with rhodamine-conjugated phalloidin (red), while cell nuclei were stained with DAPI solution (blue). Scale bar: 20 μm. A representative single slice was taken from a 3D image acquisition. (*) Indicates a statistical difference compared to the 1 mg/mL condition.

To assess cell morphology changes, F-actin was examined using confocal microscopy. As well, the size of longitudinal and transverse axes and cellular volume were determined using Icy software. At a concentration of 1 mg/mL, the average length in longitudinal axis was 49 μm and 22 μm in the transverse axis (Figure 3A), with numerous cell membrane protrusions evident throughout the cell body (Figure 3C). Upon increasing the collagen concentration to 3 mg/mL, an enlargement of the cell body was observed, with an average length in longitudinal axis to 66 μm and 14 μm in transverse axis (Figure 3A), although accompanied by a reduction in cell membrane extension (Figure 3D). Notable changes were further observed at a collagen concentration of 6 mg/mL, with a significant reduction in cell body size (average of 31 μm in the longitudinal axis and 15 μm in the transverse axis) (Figure 3A), along with a shift towards a rounder cell shape with bleb-like extensions (Figure 3E).

To deeply explore the changes in cellular size and shape observed, cell volume was determined by images segmentation and the HK-means hierarchical algorithm in Icy software. In this case, the average cell volume was 5,823 μm at 1 mg/mL, 4,622 μm at 3 mg/mL and 4,120 μm at 6 mg/mL (Figure 3B). These observations suggest that cells alter their shape in response to the density of the surrounding medium. Likewise, it is shown that cells at the maximum collagen concentration significantly reduce the extension of their longitudinal axis, although it remains greater than the transverse axis. Considering this observation, we clarify that in the rest of the text, we will refer to this change in shape as ‘rounder cells'.

**FIGURE 4:**
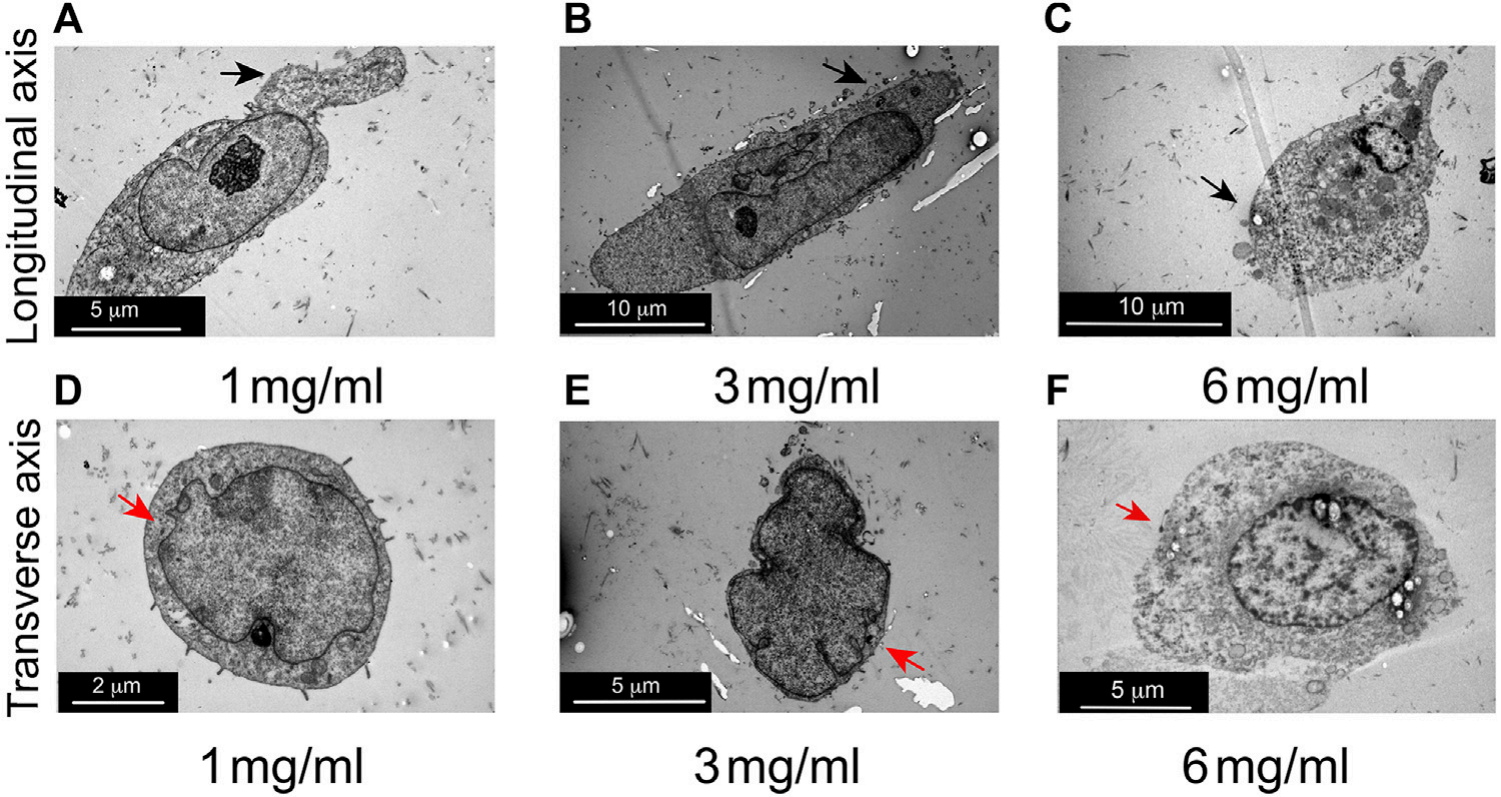
Evaluation of cell morphology in 3D growth conditions by transmission electron microscopy (TEM). **(A)** Representative longitudinal view of MDA-MB-231 cell cultured at 1 mg/mL of collagen. **(B)** Representative longitudinal view of MDA-MB-231 cells at 3 mg/mL of collagen. **(C)** Representative longitudinal view of MDA-MB-231 cells at 6 mg/mL of collagen. Notice elongate cell shape at 1 mg/mL and 3 mg/mL of collagen and rounded shape at 6 mg/mL of collagen (black arrows), as was observed by confocal laser microscopy. **(D)** Representative images of transverse view of MDA-MB-231 cells cultured at 1 mg/mL of collagen. **(E)** Representative transversal view of MDA-MB-231 cells at 3 mg/mL of collagen. **(F)** Representative transversal view of MDA-MB-231 cells at 6 mg/mL of collagen. Reduction in cytoplasmic extension as collagen concentration increases was observed, comparing cells at 3 mg/mL of collagen against cells at 1 mg/mL of collagen (red arrows). This effect is also observed in cells at 6 mg/mL of collagen (red arrows). Three images for each condition were evaluated.

To further investigate the effects of an increased concentration of collagen on cell morphology, TEM assays were conducted. Longitudinal view of MDA-MB-231 cells cultured at 1 mg/mL and 3 mg/mL of collagen, show cell membrane extensions and extended shape, as previously was observed in confocal images (Figures 4A, B). Furthermore, blebs-like extensions and rounder shape was observed in cells cultured at 6 mg/mL of collagen as previously was observed by confocal microscopy (Figure 4C). Transverse view of MDA-MB-231 cells at 1 mg/mL and 3 mg/mL of collagen, suggest a reduction in cytoplasmic volume as collagen increases (compare Figure 4D with Figure 4E). Finally, in cells seeded at 6 mg/mL, a reduction in cytoplasm was also observed (Figure 4F). The reduction in cytoplasm observed by TEM assays could explain the decrease in size demonstrated by the confocal microscopy experiments.

### 3.3 Cell migration in 3D growth conditions

Given that regulated cell deformations play a crucial role in both cell migration and invasion (Chugh and Paluch, 2018) and, considering that the cellular environment can potentially influence locomotion mechanisms (Schick and Raz, 2022), we assessed the features of cell motility of MDA-MB-231 cells under increasing density in 3D growth conditions. Our evaluation of cell migration shows that the percentage of motile cells decreases as the amount of collagen increases (Supplementary Figure S1A). For instance, at 1 mg/mL of collagen, roughly 5.9% of cells are static (see Material and Method section), compared to 45.4% for 3 mg/mL, and to 81.7% for 6 mg/mL (Table 1). Similarly, the mean instant speed of the cells decreases based on the collagen concentration present in the media (Supplementary Figure S1B). More specifically, the mean instant speed is reduced from 0.4 μm/min at 1 mg/mL of collagen to 0.3 μm/min at 3 mg/mL, and to 0.07 μm/min at 6 mg/mL (Table 1). The reduction in the number of migrating cells and their average speed in the denser collagen gel is a consequence of the increasing difficulty of movement through the environment. This assertion is supported by the α-value, or anomalous diffusion exponents. This value serves as a measure of the space explored by a random walker (in this case, the cell) as a function of time. The reference value of α = 1 corresponds to Brownian motion, where the trajectory is completely random. Cells that can move freely are expected to be superdiffusive (α>1) because the migratory patterns of cells are usually exploratory. However, we measured α-values of 0.9 for collagen concentration of 1 mg/mL, 0.6 for 3 mg/mL, and 0.3 for the concentration of 6 mg/mL (Table 1). The fact that the values are smaller than 1 indicates that the motion is subdiffusive, which is characteristic of confined environments that impede free movement. As expected, the highest concentration of collagen had the smallest α-value, promoted by the increase in collagen matrix concentration and changes in the native collagen structure, such as reduction in porosity, collagen fiber size, collagen fiber orientation, and the number of binding sites presented (Mason et al., 2013). Interestingly, despite the difference in density, cells in the medium with a concentration of 3 mg/mL had approximately the same α-value as those in 1 mg/mL, suggesting that cells were more exploratory at this higher concentration.

**TABLE 1:** Evaluation of cell migration in response to increased collagen concentrations. Videos were recorded for 24 h with a frame interval of 10 min by lens-free video microscopy. Quantitative examination of cell trajectories was tracked using the TrackMate software (Tinevez et al., 2017).

1 mg/mL	3 mg/mL	6 mg/mL
Percentage of “static” cells		
5.9	45.4	81.7
Mean instant speed (μm/min)		
0.4	0.3	0.07
α-value		
0.9	0.6	0.3
Percentage of persistence (%)		
14.2	18.0	10.4

Next, we calculated the percentage of persistence of the cells, which is the capacity to maintain the direction of motion and characterizes how straight the trajectories of cells are by comparing their net displacement to their total displacement (Supplementary Figure S1C). The percentage of persistence at 1 mg/mL is 14.2%, at 3 mg/mL is 18% and at 6 mg/mL is 10.4% (Table 1). Considering that the density of gaps in the collagen matrix directly impedes straight movement, these results suggest that the cells at 3 mg/mL exhibited much higher directionality than those at 1 mg/mL (Supplementary Figure S1C). While we computed a lower persistence value at 6 mg/mL than at 1 mg/mL, we consider the values to be close when we take into account the increased stiffness and smaller gaps of the ECM, as well as the higher number of static cells and reduction in mean instant speed (Supplementary Figure S1C compared with Supplementary Figure S1B). This suggests that the relatively “high” persistence value at 6 mg/mL could indicate a higher level of directionality, but our data are inconclusive in this respect. Based on the data, MDA-MB-231 cells might show greater persistence as collagen concentration rises, even though their migration slows down and becomes less linear at the local level, probably due to encountering more obstacles (as indicated by the α-value in Table 1). This data is consistent with previous reports showing that stiffer media or confined migration conditions enhance the migratory potential of lung cancer cells and MDA-MB-231 cells (Mathieu et al., 2016; Ishihara et al., 2023). However, due our data are not conclusive, the invasion studies in Section 3.4 will complement these observations.

### 3.4 Cell invasion in 3D growth conditions

**FIGURE 5:**
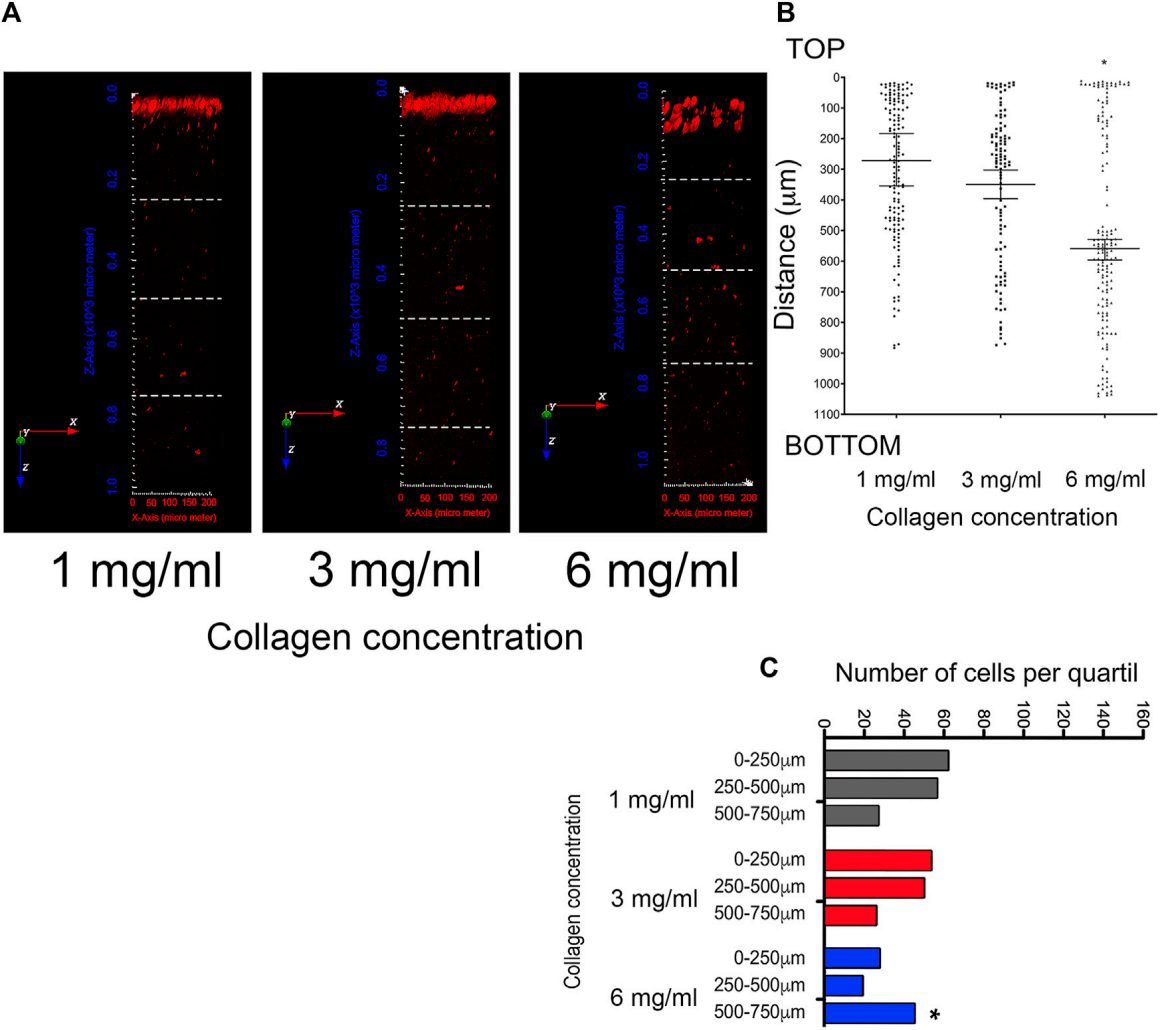
Evaluation of cell invasion in 3D growth conditions by multiphoton microscopy and second harmonic generation (SHG) technique. **(A)** Representative images of 3D projection of collagen cubes with a thickness of ∼1,000 μm at collagen concentrations of 1 mg/mL, 3 mg/mL, and 6 mg/mL. Invasive cells are indicated by red spots (stained with CellTracker Red). Three-dimensional images were reconstructed using Icy software. To quantify cell penetration into the collagen matrices, the total distance was divided into quartiles, indicated by white dashed lines **(B)** Quantification of cell invasion in the total length of collagen cube at 1 mg/mL, 3 mg/mL, and 6 mg/mL. Median ± 95% CI is shown. To mitigate potential underestimations arising from differences in collagen cube lengths, the quartile analysis considered the maximum travel distance 500–750 μm **(C)** Number of cells by quartile. (*) Indicates a statistical difference compared to the 1 mg/mL condition.

Considering the increase in persistence observed in the cell migration assays of Section 3.3, the invasion capacity within collagen at varying concentrations was evaluated. For this, fluorescently labeled cells that invaded the different media were visualized through two-photon microscopy. Figure 5A shows the 3D images reconstruction of collagen cubes with invasive cells highlighted in red. To quantify cell penetration into the collagen matrices, the total number of cells was counted across the entire length of the collagen gel (Figure 5B). To mitigate potential underestimations due to differences in collagen cube lengths, the total distance of the collagen cubes was divided into quartiles (Figure 5A). Then, the number of cells per quartile was evaluated, considering 500–750 μm as the maximum travel distance (Figure 5C). In both cases, the number of cells in each cube was quantified using Icy software.

According to this analysis, the average invasion depth was 250 μm and 350 μm for cells within collagen concentrations of 1 mg/mL and 3 mg/mL, respectively. In contrast, cells within collagen at 6 mg/mL exhibited an average invasion depth of 550 μm (Figure 5B). These data indicate that cells maintained under more dense conditions possess a higher invasion capacity, possibly facilitated by their higher persistence. Subsequently, to assess invasion capacity more effectively, a sectional analysis was conducted from the top to the 500–750 μm quartile. This analysis demonstrated that the majority of cells invading at 1 mg/mL are mainly found between 0 and 500 μm, and between 500 and 750 μm (Figure 5C). Cells contained at 3 mg/mL are distributed more evenly between 0 and 500 μm (Figure 5C). Finally, in cells maintained at 6 mg/mL of collagen, an increase in the number of cells with invasion capacity was observed in the quartile of 500–750 μm depth (Figure 5C). The data indicate that cells contained in denser matrices are more efficient at invading. This observation is supported by previous reports (Mathieu et al., 2016; Ishihara et al., 2023). Additionally, these cells show a more rounder cell shape, as well as blebs-like structures on the surface. Therefore, we propose that cells retaining the ability to migrate in dense matrices have transitioned from a mesenchymal migration mechanism to an amoeboid one.

### 3.5 Evaluation of collagen degradation and MMP secretion in 3D growth conditions

**FIGURE 6:**
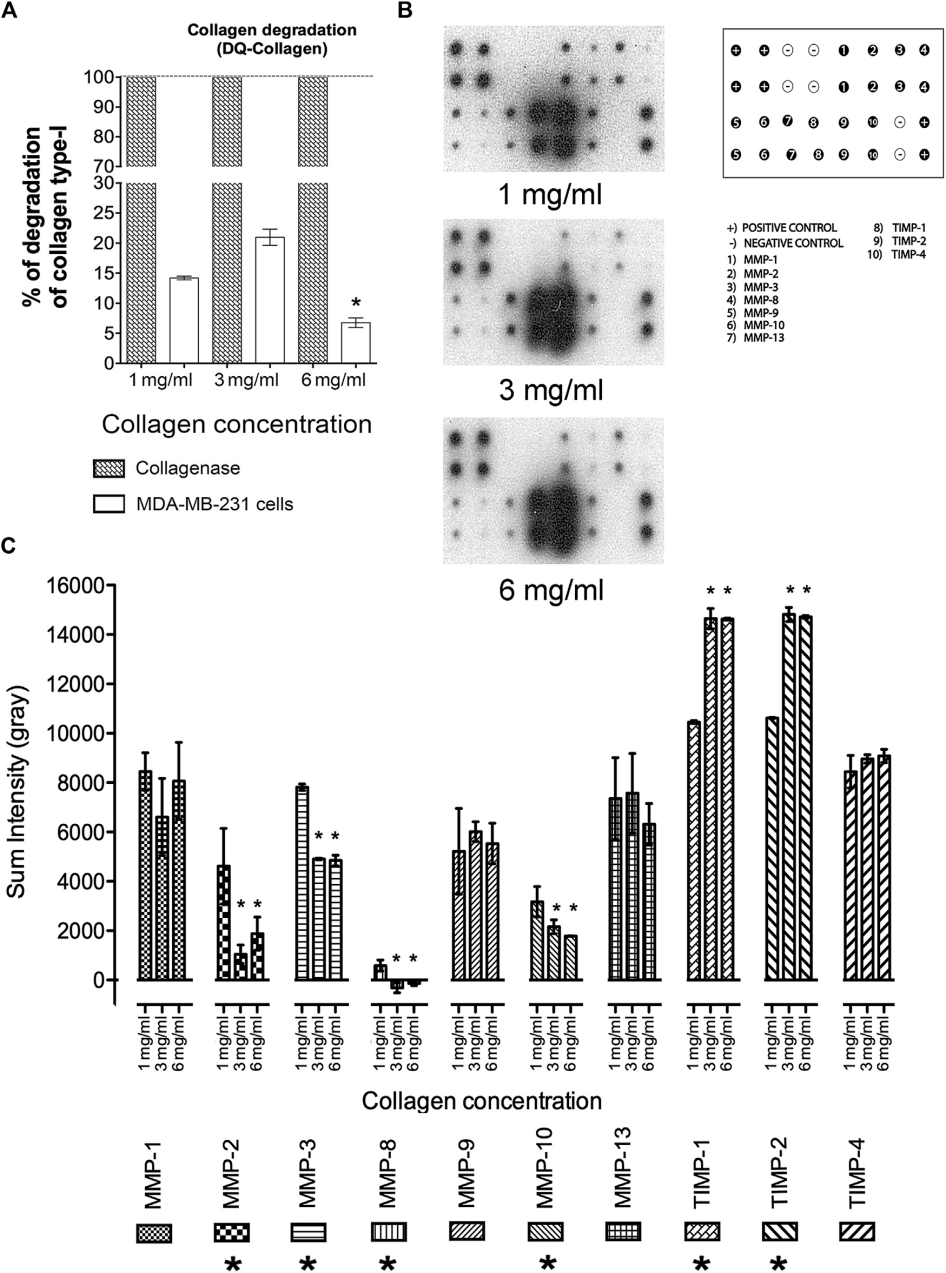
Evaluation of collagen degradation and MMP secretion in 3D growth conditions. **(A)** Percentage of collagen degradation at collagen concentrations of 1 mg/mL, 3 mg/mL, and 6 mg/mL. (*) Indicates a statistical difference compared to the 1 mg/mL condition. **(B)** Distribution of membrane arrays employed. Representative blots of membrane arrays at collagen concentrations of 1 mg/mL, 3 mg/mL, and 6 mg/mL. **(C)** Densitometric analysis of MMPs and TIMPs levels. All experiments were conducted in triplicate. (*) Indicates a statistical difference compared to the 1 mg/mL condition.

To establish whether MAT results from the 3D growth conditions across diverse collagen matrices the capacity of cells to degrade these networks was evaluated. Cells collagen degradation capacity increased with the rise in collagen concentration from 1 mg/mL to 3 mg/mL (Figure 6A). A significant reduction in collagen degradation was observed in cells maintained at 6 mg/mL of collagen, correlating with a potential shift from mesenchymal to amoeboid migration mechanisms due to a denser environment (Figure 6A). To better understand the effect of a stiffer environment on the ability to degrade the collagen matrices, the extracellular levels of several human MMPs were determined by membrane arrays (Figure 6B). Quantitative evaluation from these assays shown a reduction in the secretion levels of MMP-2, MMP-3, MMP-8, and MMP-10 as the collagen concentration increases (Figure 6C) whereas secretion of MMPs, MMP1, MMP9, MMP13 was not modified. Additionally, an increase in secretion of the tissue inhibitors of MMPs, TIMP-1 and TIMP-2 is observed in response to a stiffer medium (Figure 6C).

Based on the evidence presented, which includes the presence of a rounded morphology with bleb-like extensions, more directed migration, improved invasion capacity, and a reduction in MMP secretion, alongside an increase in TIMP-1 and TIMP-2 secretion, it can be concluded that the 3D growth conditions presented in this study, particularly with 6 mg/mL of collagen, promote MAT in a small population of MDA-MB-231 cells.

## 4 Discussion

In malignant tumors, EMT initiates the metastasis process, where cells change from an epithelial to a mesenchymal phenotype through gene expression changes. Additionally, this cell plasticity allows tumor cells to adapt to various microenvironments by switching from a mesenchymal to an amoeboid migration mode. Likewise, during BC progression, increased collagen deposition results in elevated extracellular matrix rigidity and changes in the collagen structure, such as reduction in porosity, collagen fiber size, collagen fiber orientation, and the number of binding sites presented (Mason et al., 2013). All these parameters alter cell-matrix adhesion and cell migration, and processes such as EMT, MAT, and metastasis (Wei and Yang, 2016; Omata et al., 2023). Despite the important role these changes play in the progression of metastasis in BC, there are currently no *in vitro* models that recapitulate MAT. Therefore, in the present work, our objective was to study the impact of increasingly denser environments on cell migration and invasion under 3D cell culture conditions. To recapitulate the *in vivo* environment of the breast tumor mass, MDA-MB-231 cells, classified as TNBC, were embedded in 3D collagen matrices of varying collagen concentration (1–6 mg/mL). Within these environments, it was observed that a small population of cells could enhance migration by transitioning from a mesenchymal to amoeboid phenotype-a shift probably facilitated by an elevated extracellular matrix rigidity and changes in the collagen porosity, fiber size and orientation, and the number of binding sites presented. These observations are substantiated by the following data: a transition from elongate cellular morphology to a rounder shape with bleb-like extensions, increased persistence in migration, decreased MMPs secretion and elevated levels of TIMP-1 and TIMP-2. These characteristics have previously been identified by several research groups as distinctive features of amoeboid motion mode (Wu et al., 2021). Furthermore, our findings align with *in vivo* and *in vitro* observations indicating that environments with high concentrations of ECM fibers promote increased migration and invasion (Conklin et al., 2011; Hayn et al., 2020; Morkunas et al., 2021; Sprague et al., 2021).

Through the utilization of two-photon laser scanning microscopy and SHG images, our analysis showed that higher concentrations of collagen result in a denser medium, characterized by increased stiffness, reduced extension of collagen fibers and decreased porosity, mimicking the physical environment found in malignant tumors (Fraley et al., 2015). These observations are in agreement with previous data, which showed that increasing collagen concentrations significantly decrease pore sizes and increase stiffness (Hayn et al., 2020). Notably, under these 3D conditions, the acquired amoeboid shape and migration characteristics demonstrate remarkable cell adaptability to higher density media. This is evidenced by a shift from elongated cell morphology to a rounder shape, enabling cells to navigate tight spaces (Holle et al., 2019). This is supported by our data, in which a reduction in cytoplasmic extension was observed in density conditions by confocal and TEM images, consistent with previous reports indicating that the size of the nucleus is the limiting factor for crossing dense environments (Lomakin et al., 2020). Currently, we lack data regarding the fate of the cell volume. However, we hypothesize that mechanical compression occurs. In this scenario, the high density of the medium may exert mechanical forces on the cell, leading to its compression and a subsequent reduction in overall volume. Moreover, the development of bleb-like protrusions enables cells to penetrate narrow spaces (Schick and Raz, 2022). These structures have been proven to act as leading-edge protrusions facilitating cell migration in three-dimensional environments, both *in vitro* and *in vivo* models (Paluch and Raz, 2013; Diz-Muñoz et al., 2016; Schick and Raz, 2022).

The ability of cells to adapt their migration mechanisms to diverse environmental conditions, known as migration plasticity, provides tumor cells with an advantage over normal cells for long-distance dissemination (Alexandrova et al., 2020). This finding holds importance, given that fibrous stromal tissue, a hallmark of tumors, has been demonstrated to enhance invasiveness and resistance to chemotherapy of cancer cells when they invade the stroma (Graziani et al., 2022; Omata et al., 2023). Considering the importance of MAT and the incomplete understanding of whether various matrix densities and stiffnesses promote this phenomenon *in vivo* conditions, the significance of the approach proposed by this study is of interest. Notably, all morphological changes occurred in viable cells, as demonstrated by proliferation and viability assays. Moreover, the increase in collagen matrix concentration induces changes in the native collagen structure (Mason et al., 2013), meaning that the transition to the amoeboid migration mode is likely a rapid adaptation of cells to improve their responses to environmental physical modifications.

The kinetics of cell migration at higher concentrations of collagen were affected by the fact that cells in a 3D environment need to resist external forces across a larger surface area (Tibbitt and Anseth, 2009). At low collagen concentrations, the instantaneous speed of the cells was higher, while at the highest concentration, a greater number of cells were static, likely due to being trapped in the matrix. The subdiffusive cell motion indicates that this is a consequence of the constraints imposed by media with a higher density of collagen, which yield matrices that are stiffer with reduction in porosity, fiber size and fiber orientation among other factors (Mason et al., 2013). However, our analysis suggests that the cells are more persistent in media with higher collagen concentrations. This was confirmed by the persistence measurements, which had a higher value for cells in the 3 mg/mL condition than in the 1 mg/mL one, despite the higher difficulty of maintaining a straight trajectory through denser collagen. On the other hand, cells in the 6 mg/mL condition showed a “high persistence,” relatively speaking. However, it was difficult to establish an exact quantitative comparison due to the much higher number of static cells and the much denser medium. To explore this question further, we looked to decouple these factors. One way was the invasion study, where we measured the penetration of cells in the different media without factoring in the time-evolution component. Our data showed that cells stimulated to invade substrates of 6 mg/mL could infiltrate the matrix to a greater distance compared to cells contained at 1 mg/mL and 3 mg/mL of collagen. In addition, cells in higher concentrations were rounder and exhibited more bleb-like structures. Based on all this information, we concluded that the cells contained in the denser gels exhibited amoeboid migration. These observations align with previous reports indicating that amoeboid migration has consistent migration rates (Wyckoff et al., 2000; Condeelis and Segall, 2003), with minimal interaction with the ECM (Yamada and Sixt, 2019). They also underscore the usefulness of our model in studying the MAT process.

On the other hand, it has been reported that amoeboid migration is independent of ECM proteolysis (Yan et al., 2016). Considering the significance of reduced ECM degradation as a distinctive characteristic of amoeboid migration, we evaluated the ability of MDA-MB-231 cells to degrade collagen and evaluate the secretion of various MMPs under the diverse experimental conditions employed in this study. According to the data obtained, increasing collagen concentration from 1 mg/mL to 3 mg/mL enhances collagen degradation capacity. Taking into account this observation and the above-mentioned data, including the significant cell membrane protrusions observed, as well as an elongate cell shape, we propose that MDA-MB-231 cells exhibit a mesenchymal migration pattern under 1 mg/mL and 3 mg/mL, which is dependent on extracellular matrix degradation. In contrast, cells cultured at 6 mg/mL of collagen exhibit a significant reduction in their ability to degrade collagen. Based on this information and the data presented earlier, we conclude that MDA-MB-231 cells cultured at the maximum collagen concentration undergo a transition in their migration mechanism from mesenchymal to amoeboid, independent of extracellular matrix degradation.

A more exhaustive evaluation of MMPs secretion during amoeboid migration reveals that an increase in collagen concentration leads to a gradual reduction in the levels of MMP-2, MMP-3, MMP-8, and MMP-10. Conversely, under the same experimental conditions, a gradual increase in TIMP-1 and TIMP-2 secretion was observed. Based on this data, we propose that in our experimental conditions, there is an augmentation in TIMP function during amoeboid migration, resulting in a reduction in MMPs activity. This observation is supported by previous studies demonstrating the negative regulatory role of TIMPs in MMPs (Peeney et al., 2022).

Likewise, MMPs have been identified as the primary proteinases associated with tumor development. Initially recognized for their role in promoting tumor progression by modifying the extracellular matrix through proteolysis, accumulating evidence suggests that certain MMPs also play protective roles in cancer progression. Moreover, the same MMP may exhibit conflicting functions depending on the specific cell type or cancer stage (Kwon, 2023). However, the potential impact of the decrease in MMPs and the increase in TIMPs during amoeboid migration is a matter for further research. Nonetheless, it is crucial to emphasize that in our 3D model the decrease in MMPs activities correlates with the observed increase in their inhibitors, which is here proposed as a new feature of amoeboid migration.

Different studies have shown how the use of various substrates promotes amoeboid migration (Heit and Kubes, 2003; Chikina and Alexandrova, 2018). However, the 3D culture conditions presented in this study demonstrate how the increase in collagen concentration employed as ECM can recapitulate the amoeboid migration process observed *in vivo*. This point is significant considering that the amount of collagen used correlates with the levels of this protein present in healthy breast tissues and malignant tumors. Therefore, we propose that the cellular and functional assays presented could recapitulate not only the main characteristics of amoeboid movement but also its association with physiological processes. For these reasons, our method could be an excellent system to consistently explore the physiology of amoeboid migration. Considering this, we propose the use of our 3D conditions to address some questions. For example, defining which cells can undergo MAT or, evaluating the role of MMPs and TIMPs during amoeboid migration, etc. Thus, the use of the tools outlined in this study could contribute to understanding these and other associated processes.

In conclusion, we demonstrate that MDA-MB-231 cells contained at 6 mg/mL of collagen, undergo amoeboid migration, as is evidenced by changes in morphology, increasing in percentage of persistence in movement, greater invasion capacity, decreasing in MMP secretion, and increasing in TIMP levels.

## Data Availability

All the original contributions from the study are included in the article and the Supplementary Material. Requests for access to the datasets should be directed to AA-R at a_aguilar@unam.mx.

## Supplementary material

SUPPLEMENTARY FIGURE S1
**(A)** Example of cell migration tracks of MDA-MB-231 cells within collagen matrices at concentrations of 1 mg/mL, 3 mg/mL, and 6 mg/mL. Cells were recorded over 24 hours with a frame interval of 10 min using a lens-free microscope. There is observed a reduction in the number of cells capable of migrating as the collagen concentration increases. Similarly, a decrease is observed in the distance at which the cells move with increasing collagen concentration. To improve data presentation, a subset of only 15 cells tracks were randomly chosen for each concentration. **(B)** Box-and-whisker plots of velocity. There is no overlap between the upper and lower quartiles of cells contained in collagen at 1 mg/mL and 6 mg/mL. This discrepancy suggests significant differences among the data (*n* = 135 at 1 mg/mL, *n* = 81 at 3 mg/mL and *n* = 71 at 6 mg/mL). **(C)** Box-and-whisker plots of persistence. There is overlap between the upper and lower quartiles across cells contained at the different collagen concentrations. This indicates that, although the mean differs among samples, at least 50% of the persistence data at 1 mg/mL, 3 mg/mL, and 6 mg/mL collagen concentrations are similar (*n* = 135 at 1 mg/mL, *n* = 81 at 3 mg/mL and *n* = 71 at 6 mg/mL).
