# Supplementary figures and images for: Three-dimensional cell culture conditions promoted the Mesenchymal-Amoeboid Transition in the Triple-Negative Breast Cancer cell line MDA-MB-231
Source: Front Cell Dev Biol. 2024 Aug 2;12:1435708. doi: 10.3389/fcell.2024.1435708 (PMC11327030; doi:10.3389/fcell.2024.1435708)

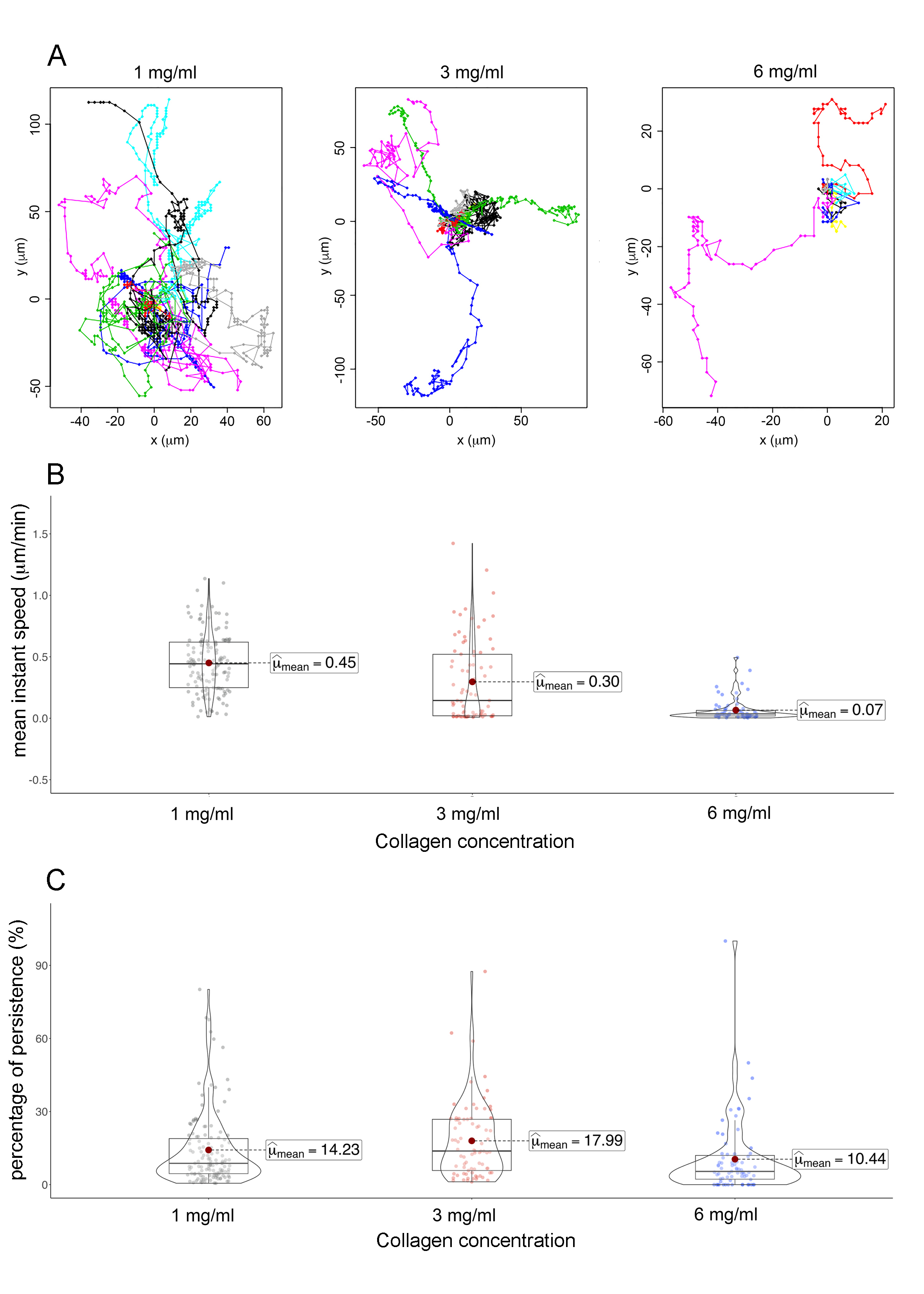

Supplement: Supplementary file 1 [file Image1.JPEG]
